# Optimizing a linear ‘Doggybone’ DNA vaccine for influenza virus through the incorporation of DNA targeting sequences and neuraminidase antigen

**DOI:** 10.1093/discim/kyad030

**Published:** 2024-01-03

**Authors:** Robert F Cunliffe, David C Stirling, Ilaria Razzano, Valarmathy Murugaiah, Emanuele Montomoli, Sungwon Kim, Madina Wane, Helen Horton, Lisa J Caproni, John S Tregoning

**Affiliations:** Department of Infectious Disease, Imperial College London, London W2 1PG, UK; Department of Infectious Disease, Imperial College London, London W2 1PG, UK; Department of Life Sciences, University of Siena, 53100 Siena, Italy; VisMederi srl, Siena, 53100, Italia; Department of Infectious Disease, Imperial College London, London W2 1PG, UK; VisMederi srl, Siena, 53100, Italia; Department of Molecular and Developmental Medicine, University of Siena, 53100 Siena, Italy; Touchlight Genetics Ltd, Hampton, TW12 2ER, UK; Touchlight Genetics Ltd, Hampton, TW12 2ER, UK; Touchlight Genetics Ltd, Hampton, TW12 2ER, UK; Touchlight Genetics Ltd, Hampton, TW12 2ER, UK; Department of Infectious Disease, Imperial College London, London W2 1PG, UK

**Keywords:** DNA, vaccines, influenza, Neuraminidase

## Abstract

Influenza virus represents a challenge for traditional vaccine approaches due to its seasonal changes and potential for zoonotic transmission. Nucleic acid vaccines can overcome some of these challenges, especially through the inclusion of multiple antigens to increase the breadth of response. RNA vaccines were an important part of the response to the COVID-19 pandemic, but for future outbreaks DNA vaccines may have some advantages in terms of stability and manufacturing cost that warrant continuing investigation to fully realize their potential. Here, we investigate influenza virus vaccines made using a closed linear DNA platform, Doggybone™ DNA (dbDNA), produced by a rapid and scalable cell-free method. Influenza vaccines have mostly focussed on Haemagglutinin (HA), but the inclusion of Neuraminidase (NA) may provide additional protection. Here, we explored the potential of including NA in a dbDNA vaccine, looking at DNA optimization, mechanism and breadth of protection. We showed that DNA targeting sequences (DTS) improved immune responses against HA but not NA. We explored whether NA vaccine-induced protection against influenza virus infection was cell-mediated, but depletion of CD8 and NK cells made no impact, suggesting it was antibody-mediated. This is reflected in the restriction of protection to homologous strains of influenza virus. Importantly, we saw that including both HA and NA in a single combined vaccine did not dampen the immune response to either one. Overall, we show that linear dbDNA can induce an immune response against NA, which may offer increased protection in instances of HA mismatch where NA remains more conserved.

## Graphical Abstract

**Graphical Abstract:**
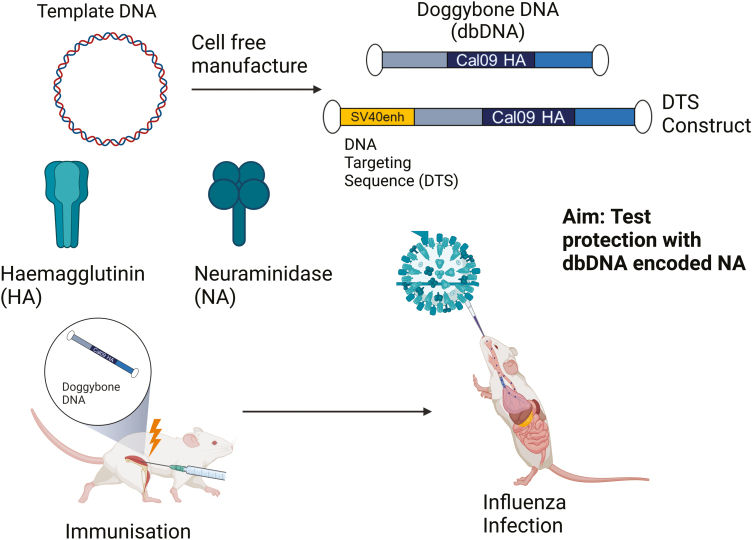

## Introduction

In the absence of universal vaccines that can protect against all influenza viral variants, faster manufacturing processes are needed for influenza vaccines; this would be beneficial for both seasonal/ endemic and pandemic strains. Seasonal vaccines for the influenza virus need to be reformulated on an annual basis to confer protection against mutations that arise in the surface-exposed haemagglutinin (HA) and neuraminidase (NA) proteins [1]. Pandemic readiness is also compromised since current manufacturing processes can take at least 6 months to produce sufficient quantities to protect the population [2]. Novel cassette-based, nucleic acid vaccine platforms proved themselves to be highly valuable during the COVID-19 pandemic [3] and can potentially be applied to influenza vaccines [4]. The pandemic demonstrated the need for a diverse range of vaccine platforms to accelerate testing and to increase production; therefore there is value in developing alternatives and having more shots on goal. DNA vaccines are a promising vaccine platform, that can be rapidly produced and have a potential cost and temperature stability benefit over other platforms [5, 6].

Traditionally, DNA vaccines targeting influenza virus have taken the form of circular supercoiled plasmids which encode the sequence of an influenza viral protein [7]. Such vaccines have been shown to be immunogenic in multiple species, and humans, generating protective immunity against influenza viral challenge [8]. Doggybone DNA (dbDNA) represents the first of a kind rapidly produced, bacterial sequence-free construct; a huge advantage of this platform is that it is synthesized through an enzymatic process that avoids the need for bacterial fermentation [9]. The resulting DNA construct is a linear vector cassette containing the encoded antigen sequence of interest, promoter, poly A tail, and closed, fully complementary ends. First-generation dbDNA has been shown to induce similar levels of cellular and humoral immunity when compared to plasmid DNA against HIV [10], influenza [9, 10], and HPV [11] in mice.

However, further optimization is required to overcome the challenges that DNA vaccines have had moving from mice into large animal models and humans [12]. One consideration is to improve the expression of the antigen encoded on the DNA vaccine construct. Compared to RNA vaccines, DNA faces a double barrier to expression, as it needs to enter the nucleus for transcription to occur before the antigen can be translated. Entry of DNA into the nucleus is particularly a barrier in the case of mitosis-inactive cells such as antigen-presenting cells (APCs) and muscle cells [12]. Incorporation of DNA targeting sequences (DTS) into DNA plasmids can facilitate enhanced import of foreign DNA into the non-dividing nucleus [13] and therefore improve antigen expression. These DTS are known to bind transcription factors, which, via their Nuclear Localisation Signal (NLS) sequences, help shuttle the DNA into the nucleus. For example, the inclusion of SV40 enhancer-derived DTS into plasmid DNA was found to enhance nuclear localization [14].

As well as the speed of production, another advantage of nucleic acid vaccines is the ability to incorporate multiple different antigens. The predominant influenza virus surface glycoprotein haemagglutinin (HA) mediates host cell binding and entry [15], but also drives strain drift. Currently, licensed inactivated influenza virus vaccines are designed to induce anti-HA antibodies as these, when measured as haemagglutination inhibition (HAI) titres, are accepted correlates of protection [16]. Compared to HA less attention has been paid to neuraminidase (NA) as an antigen, but it could play an important role in improved influenza vaccines [17]. NA plays a key role in the life cycle of the influenza virus by cleaving terminal sialic acids from infected host cells, allowing the subsequent release of nascent virus particles from the cell [18]. High-dose, split virion, inactivated influenza vaccines induce more anti-NA responses in the elderly [19] with a suggestion that this could improve protection, though there are still several questions that need addressing about its inclusion and the optimal platform used to deliver it [17]. One important question in the design of vaccines is how NA might provide protection. Anti-NA antibodies do not prevent viral entry, instead, they limit the spread of the virus, contributing to immunity against influenza in animal models [20, 21]. This is supported by data from human challenge studies conducted in the 1970s, which revealed that anti-NA antibody titres inversely correlated with both viral shedding and observed symptoms [22]. However, the inclusion of NA may also provide a boost to cell-mediated killing of infected cells by the inclusion of more epitopes under less selection pressure than HA; this could be important when considering vaccine platforms.

The aim of the current study was to improve a dbDNA vaccine against influenza virus through modifications in the non-coding sequences of the DNA and the inclusion of NA. We compared the inclusion of DTS on dbDNA encoding HA or NA. We observed that the inclusion of the SV40 enhancer (contains two tandem copies of DTS) was advantageous for HA, improving protection even at a suboptimal dose. We saw no impact of DTS on dbDNA encoding NA, but the vaccine was partially protective against homologous virus challenge. To understand more the impact of including NA in an influenza vaccine, we sought to investigate unanswered questions relating to anti-NA immunity, in particular the breadth of protection and whether protection arising from NA immunization operated through cell-mediated killing. NK and CD8 T cell depletion studies indicated that NA protection was antibody-dependent and not strongly dependent on cell-mediated responses. Studies using heterologous strains suggest that dbDNA-encoded NA immunization only provides protection against closely matched strains. This study supports the inclusion of NA in nucleic acid vaccines, suggesting they can increase the breadth of response through antibody-mediated protection.

## Materials and methods

### Doggybone (dbDNA) construction

The amino acid sequences of influenza A virus (H1N1) strain (A/California/07/2009) haemagglutinin (HA) and neuraminidase (NA) surface proteins were codon-optimized, synthesized and cloned into proTLx-K D3F2 plasmid (including CMV promoter and enhancer, 5ʹ-UTR and SV40 late polyadenylation signal) with or without a SV40 enhancer sequence (containing two tandem 72bp DTS sequences) upstream of the CMV promoter/enhancer [23]. The resulting pDNA template comprised the expression cassette flanked by two recognition/binding sites for the TelN pro-telomerase from *E. coli* phage N15. dbDNA was produced as previously described [10]. Briefly, the template plasmid was denatured using NaOH and then quenched in the reaction buffer containing custom primer, dNTPs, Phi29 polymerase and pyrophosphatase. Upon mixing, the reaction was incubated at 30°C for 30 h. Concatemeric DNA was processed by the addition of TelN protelomerase, BamHI and Exonuclease III. The digest mixture was cleaned from reaction components and precipitated using polyethylene glycol (PEG) 8000.

### Mouse immunization and influenza infection

Six- to eight-week-old female BALB/c mice were obtained from Charles River Ltd (Doncaster, UK) and kept in specific-pathogen-free (SPF) conditions in accordance with the United Kingdom’s Home Office guidelines and animal care procedures followed recommendations from appropriate committees. All work was approved by the Animal Welfare and Ethical Review Board (AWERB) at Imperial College London; work was licensed under P4EE85DED. Mice were anaesthetized using isoflurane prior to being injected with 50 μl i.m. dbDNA; a dose of dbDNA described in individual studies. Following immunization, the injection site was electroporated (EP) using electrodes connected to an ECM 830 square-wave electroporation system (BEX, Japan). Pulses consisted of 150 V of positive and negative polarity at a rate of 1 pulse/s, with each pulse lasting 50 ms. Mice were immunized in a prime only or prime boost regime (with injections 4 weeks apart). For infections, mice were anaesthetized using isoflurane followed by i.n. application with 2000 PFU A/California/07/2009 (H1N1) influenza virus; 1500 PFU A/Puerto Rico/8/1934 or 20,000 PFU X31 in a 100 µl volume as previously described [24]. Group sizes were selected based on previous studies on immune responses to HA-based vaccines, where a 10-fold change in antibody response gave a significant difference in protection [25]; in the same strain of mice, using G*power software [26], *n* = 4 per group gives a power of 0.98.

### Tissue and cell isolation

At specified time points after immunization, blood samples were taken by tail vein bleed, and sera was isolated following clotting by centrifugation. Mice were culled using intraperitoneal pentobarbitone (20 mg dose, Pentoject, Animalcare Ltd. UK), and tissues were collected as previously described [24]. Blood was collected from carotid vessels, and sera was isolated after clotting by centrifugation. If necessary, the left lobe of the lung was removed and frozen at −80°C for later viral load analysis. Spleens were homogenized by passage through 100 μm cell strainers, then centrifuged at 300 × *g* for 5 min. Supernatants were removed, and the cell pellet was treated with red blood cell lysis buffer before centrifugation at 300 × *g* for 5 min. The remaining cells were resuspended in RPMI 1640 medium with 10% FBS, and viable cell numbers were determined by trypan blue exclusion.

### ELISA

Antibodies specific to influenza H1N1 virus were measured in sera using a semiquantitative ELISA [27]. MaxiSorp 96-well plates (Nunc) were coated with antigen at 1 µg/ml either HA (Sino Biological) or NA (Sino Biological) in 1× PBS, or a combination of anti-murine lambda and kappa light chain-specific antibodies (Novus Biologicals) for standard wells and incubated overnight at 4°C. Plates were blocked with 1% BSA in PBS. Following the addition of immunization sera or a serially diluted mouse IgG (Southern Biotech) as a standard to quantify specific antibodies, bound IgG was detected using HRP-conjugated goat anti-mouse IgG (Abcam). TMB (Thermo Fisher) with 2N H_2_SO_4_ as a stop solution was used to detect the response and absorbance read at 450 nm.

### Haemagglutination inhibition assay (HAI) assay

Serum samples were pre-treated with Receptor Destroying Enzyme (RDE, Denka Seiken) for 18 h at 37°C before inactivating the enzyme at 56°C for 1 h. RDE-treated serum was 2-fold serially diluted across a V-bottom plate (Merck) with 1X PBS and incubated with pre-diluted 4 haemagglutinating units of A/California/07/2009 (H1N1) virus per well for 30 min at room temperature. 50 μl of 1% turkey erythrocytes (Envigo) diluted in PBS was then added to each well, and the plate was incubated for 30 min at 4°C before scoring the response.

### Lentiviral neuraminidase inhibition enzyme-linked lectin assay (ELLA)

Assay as described elsewhere [28]. Briefly, 96-well Nunc MaxiSorp plates were coated with 25 µg/ml fetuin (Sigma) in 1× coating buffer (KPL). Serum samples that had been heat-inactivated at 56°C for 60 min were serially diluted 2-fold (starting dilution 1:10) across the plate in sample buffer (1% BSA, 0.5% Tween 20 in PBS with 0.9 mM CaCl_2_, 0.5 mMMgCl_2_, Sigma). A standard dilution of H11N1 pseudovirus (N1 from A/California/07/2009 strain) was pre-determined as the 90% maximal effective concentration and added to each well. Plates were incubated for 18 h at 37°C with 5% CO_2_. Virus without serum served as virus control and sample buffer only as negative control. After incubation, 1 µg/ml peanut agglutinin-HRP (Sigma) was added. Signals were developed with TMB solution (Thermo Fischer) for 5 min, stopped with 0.5 M HCl, and read at 450 nm. NI (neuraminidase inhibition) titres were defined as the reciprocal of the highest serum dilution at which the mean absorbance results in at least 50% inhibition of the virus control mean signal.

### T cell ELISpot

IFN-γ ELISpot assays were performed using a commercial kit from Abcam (ab64029) following the manufacturer’s recommendations. Cells were stimulated with 1.25 μg/ml anti-CD28 (Clone 37.51, BD) and premade 15-mer sequences with 11 amino acids overlap peptides from influenza A (H1N1) HA (Peptivator, Miltenyi Biotech) or NA (PepMix^TM^, JPT). Spots were counted using the AID iSpot reader and ELISpot Reader software V 7.0.

### Influenza viral load

Viral load *in vivo* was assessed by Trizol extraction of RNA from frozen lung tissue disrupted in a TissueLyzer (Qiagen. RNA was converted into cDNA, and quantitative PCR was carried out using 0.1 μM forward primer (5ʹ-AAGACAAGACCAATYCTGTCACCTCT-3ʹ), 0.1 μM reverse primer (5ʹ-TCTACGYTGCAGTCCYCGCT-3ʹ), and 0.2 μM probe (5ʹ-FAM-TYACGCTCACCGTGCCCAGTG-TAMRA-3ʹ) for the influenza M gene on a Stratagene Mx3005p (Agilent technologies). M-specific RNA copy number was determined using an influenza M gene standard plasmid [24].

### NK cell or CD8 depletion

Mice were i.p. injected with 500 μg Anti-Mouse CD8a antibody (clone 53-6.7, AssayGenie) (500 μl of PBS for control mice in the CD8 depletion study) or 50 μg Anti-Asialo GM1 Polyclonal Antibody (Invitrogen) 24 h before being intranasally infected with influenza. Mice were i.p. injected again with the same antibodies on day 2 post-infection.

### Statistical analysis

Calculations as described in figure legends were performed using Prism 9 (GraphPad Software, USA).

### Data availability

Numerical data from graphs are available on request to the corresponding author.

## Results

### Including DNA targeting sequences (DTS) in dbDNA improves immunogenicity to influenza HA

**Figure 1:**
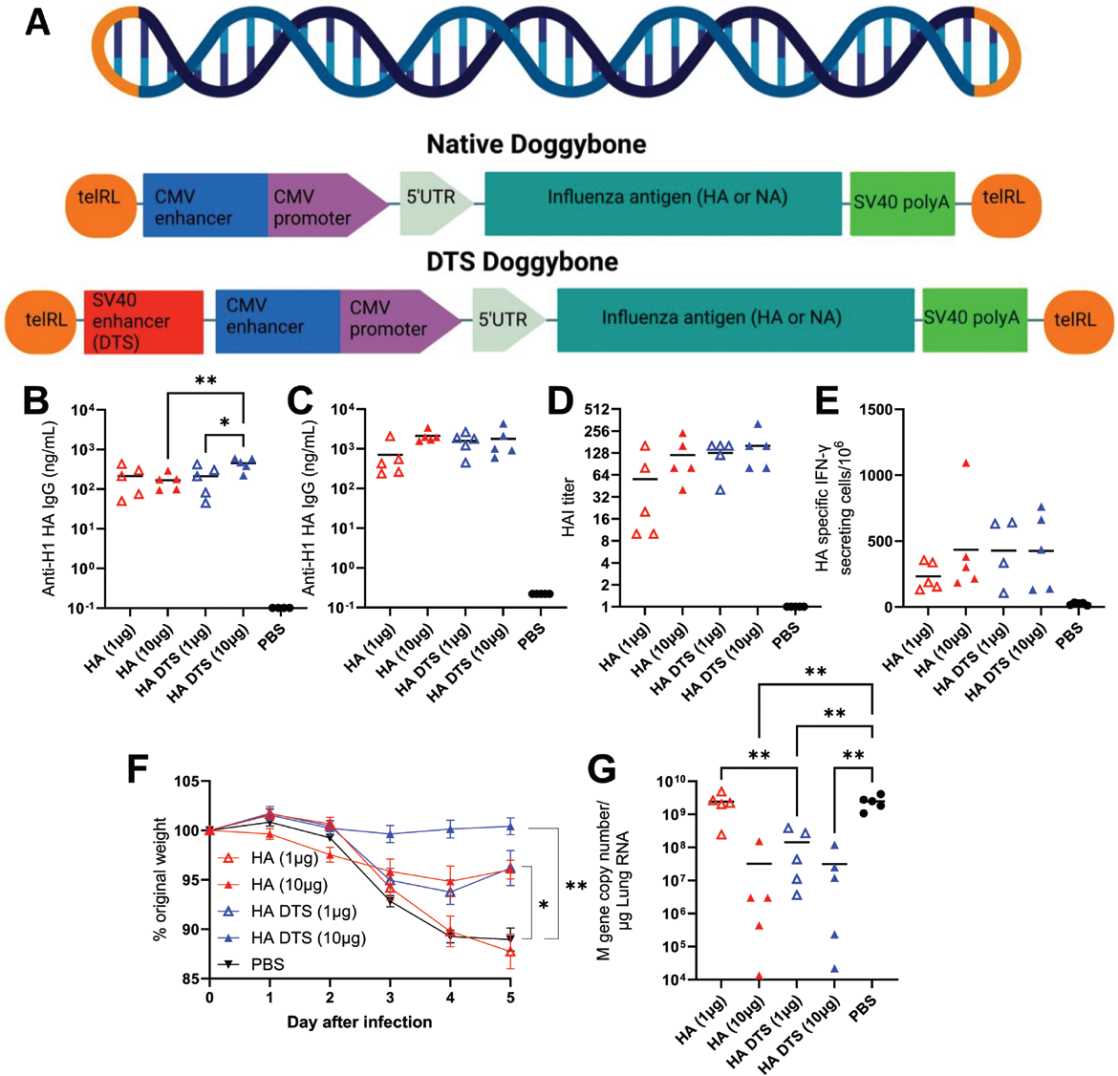
**HA-encoding dbDNA with DTS confers increased protection against influenza challenge.** Structure of the dbDNA with or without DNA targeting sequences (DTS) (**A**). Female BALB/c mice were intramuscularly (IM) immunized with 1 or 10 µg Doggybone DNA (dbDNA) encoding for Haemagglutinin (HA) in a prime-boost regime at 0 and 4 weeks. The dbDNA constructs were with or without DTS. Blood was collected for analysis of HA-specific antibody 4 (**B**) and 6 (**C**) weeks after the start of the study. Haemagglutination inhibition (HAI) titre was assessed at the 6-week timepoint (**D**) Spleens were collected and assessed for HA-specific T cells by ELISpot (**E**). In a separate study, mice were infected intranasally with influenza virus 4 weeks after a single vaccination dose. Weight loss was measured after infection (**F**), and viral load in the lung was measured 5 days after infection (**G**). *N* = 5 mice per group, points represent individual animals, and lines represent means. *P < 0.05, **P < 0.01. Study performed once. Statistical analysis was performed by ANOVA with Tukey test. Doggybone image drawn using BioRender.

The dbDNA platform, because it is made through a cell-free process, offers considerable manufacturing advantages. It is a capped linear DNA construct (Fig. 1A). The overall aim was to improve upon earlier dbDNA vaccines against influenza [10] through altering non-coding DNA or the inclusion of NA. We initially investigated whether the inclusion of DNA targeting sequences (DTS) would improve the immunogenicity of dbDNA vaccines. Two versions of dbDNA vaccines encoding the HA antigen from the H1N1 A/California/07/2009 strain of influenza were produced with and without the SV40 enhancer (containing 2xDTS sequences). BALB/c mice were immunized with either 1 or 10 µg of vaccine, delivered intramuscularly with electroporation. Immunogenicity was assessed following a prime-boost regimen, with a 4-week interval. Two dose levels were used, high and low, as we have previously noted that high doses of vaccines in mice can mask subtle effects on immunogenicity [25]. All mice immunized with HA-encoding dbDNA had detectable anti-HA antibody titres (Fig. 1B). After prime, HA DTS induced significantly higher HA-specific antibody titres compared to HA at the 10 µg dose (*P* < 0.01, Fig. 1A). In addition, a dose-dependent effect was seen for immunization with the DTS construct, with a 10-µg immunization inducing significantly higher titres than 1 µg (*P* < 0.05, Fig. 1A). There was no significant difference in antibody titre following booster (Fig. 1C). A similar trend was seen in the HAI titres after boost (Fig. 1D); all groups gave an HAI titre over 40. Likewise, there were slightly more HA-specific T cells in the DTS groups than the no DTS comparator, but this was not significant (Fig. 1E).

In a separate study, we tested whether incorporation of DTS improved protection efficacy against influenza infection. Following a single immunization, mice were intranasally infected with 2000 PFU A/California/07/2009 H1N1 influenza virus and weight change was measured as a readout of disease severity. The combination of high viral titre for the challenge and relatively low vaccine dose was used to enable differences in protection to be observed. Mice immunized with 10 µg HA DTS did not lose any weight after infection (Fig. 1F), with a significant level of protection compared to the PBS group (*P* < 0.001). Similarly, mice immunized with 10 µg HA lost significantly less weight than the PBS control group at d5 (*P* < 0.001), though there was some weight loss on days 3–5 compared to starting weight. The 1 µg HA DTS group had a similar weight change trajectory as the 10 µg HA alone group, with partial protection (*P* < 0.05); this was better than 1 µg HA alone, which was not significantly different to PBS group. There was no significant difference in viral load between the PBS and 1 µg HA alone groups, indicating it offered no protection against disease or infection (Fig. 1G). 1 µg HA DTS also gave greater protection against viral infection with significantly less viral RNA recovered from lungs on day 5 after infection than the 1 µg HA alone group (*P* < 0.001). The other groups had a significantly lower viral load (*P* < 0.001). Interestingly, the protection conferred by 1 µg HA DTS was comparable to that from 10 µg HA alone. These data indicate that the inclusion of the DTS sequences within the SV40 enhancer may be beneficial for dbDNA-encoded HA antigen vaccines.

### Immunization with dbDNA encoding NA is immunogenic at higher doses and partially protective against infection

**Figure 2:**
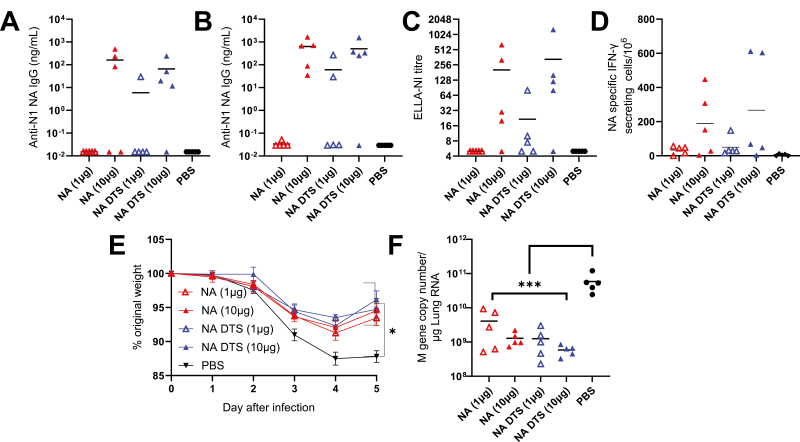
**dbDNA encoded NA is immunogenic and partially protective against infection.** Female BALB/c mice were intramuscularly immunized with 1 or 10ug dbDNA encoding Neuraminidase (NA), in a prime-boost regime at 0 and 4 weeks. Blood was collected for analysis of HA-specific antibody 4 (**A**) and 6 (**B**) weeks after the start of the study. Enzyme-linked lectin assay (ELLA) assay for NA-inhibiting antibody titres was performed using serum collected 6 weeks after the start of the study (**C**). Spleens were collected and assessed for NA-specific T cells by ELISpot (**D**). In a separate study, mice were infected intranasally with influenza virus 6 weeks after prime-boost vaccination. Weight loss was measured after infection (**E**), and viral load in the lung was measured 5 days after infection (**F**). *N* = 5 mice per group, points represent individual animals, and lines represent means. *P < 0.05, **P < 0.01, ***P < 0.001. Study performed once. Statistical analysis was performed by ANOVA with Tukey test.

An additional approach to broaden protection for an influenza vaccine is to include additional antigens. Targeting the other viral surface antigen, Neuraminidase (NA) has been shown to be beneficial, to test this, we explored the immune response to dbDNA encoded NA. dbDNA encoding the NA antigen from the H1N1 A/California/07/2009 strain was made with and without DTS. BALB/c mice were immunized with either 1 or 10 µg of vaccine intramuscularly with electroporation and immunogenicity assessed following a prime-boost regimen. Only immunization with 10 µg of either dbDNA construct elicited anti-NA IgG antibodies, and the difference in titres between constructs was not significant (Fig. 2A and B). To determine the function of the antibodies, we used a pseudotype-based ELLA assay [29]. The ELLA results correlated with ELISA results, showing a dose response and higher titre in the 10 µg groups, with more responders (3/5) in the 1 µg NA DTS group than NA (0/5, Fig. 2C). Only the 10 µg dose groups induced NA-specific T cells with no significant difference in the presence and absence of DTS (Fig. 2D).

In a subsequent study, protection against infection following two doses of dbDNA was tested. When challenged with 2000 PFU A/California/07/2009 influenza virus, all immunized mice were partially protected and recorded significantly less weight loss compared to the PBS control at day 5 after infection (*P* < 0.05, Fig. 2E). The viral load in the lungs of mice immunized with either dbDNA construct was significantly lower than the PBS control group 5 days-post infection regardless of immunization dose (*P* < 0.01, Fig. 2F). These data show that DNA-encoded NA is immunogenic and can provide partial protection against influenza infection and disease.

### Immune response protection provided by NA immunization is not CD8 T or NK cell mediated

**Figure 3:**
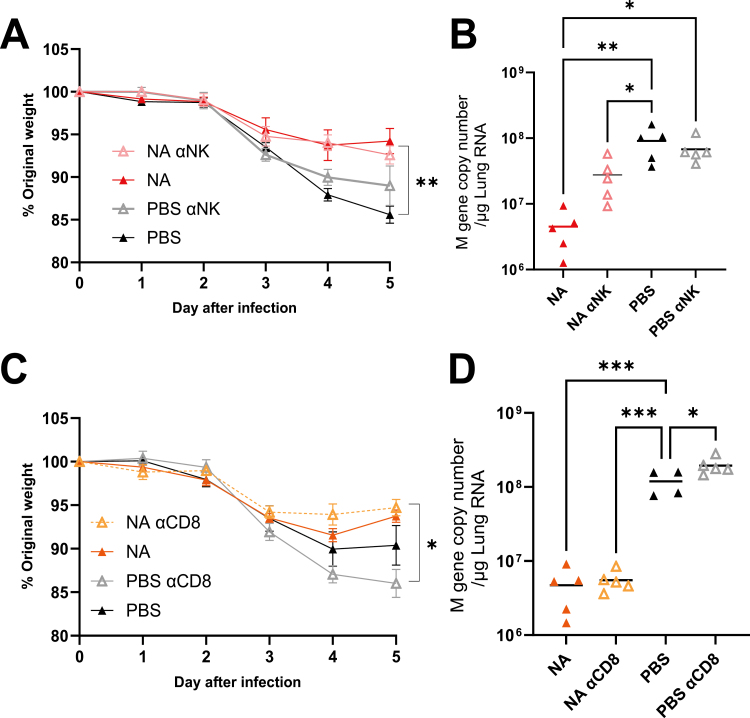
**NK and CD8 T cells are not necessary for protection following NA-dbDNA immunization.** Female BALB/c mice were intramuscularly immunized with 10 µg dbDNA encoding NA at 0 and 4 weeks. NK or CD8 cells were depleted by intraperitoneal (IP) administration of either anti-NK (Anti-asialo GM1: **A-B**) or anti-CD8 (**C-D**) specific antibody during intranasal infection with influenza virus. Weight loss was measured after infection (A and C), and viral load in the lung was measured 5 days after infection (B and D). *N* = 5 mice per group, points represent individual animals, and lines represent means. Study performed once. *P < 0.05, **P < 0.01. Statistical analysis was performed by ANOVA with Tukey test.

Whilst HA-mediated protection against infection is strongly associated with neutralizing antibody, there are many knowledge gaps still to be addressed about NA-mediated protection. Understanding the mechanism by which NA induces protection is important in the selection of vaccine platform, as some vaccines favour antibody over cell-mediated responses, for example, modified mRNA [25]. We hypothesized that there might be a role in the killing of infected cells by antigen-specific CD8 T cells or antibody-dependent NK cells. To test this, we looked at the effect of depleting NK and CD8 T cells in mice immunized with NA-encoding dbDNA to test these hypotheses. Mice were primed and boosted with dbDNA expressing NA-DTS, and the NA-specific IgG ELISA confirmed that the immunization was immunogenic. Mice were then infected with influenza virus, with and without NK cell depletion using anti-Asialo GM1 (depletion confirmed by flow cytometry [Supplementary Fig. S1]). The immunized mice lost significantly less weight on day 5 than control mice (*P* < 0.01, Fig. 3A). Both immunized groups had significantly lower viral load than the unimmunized mice (*P* < 0.05, Fig. 3B). However, there was no difference between the NK depleted and control immunized mice. The effect of NK cell depletion on influenza infection was compared in control, unvaccinated mice. Surprisingly, there was no difference in weight loss (Fig. 3A) or viral load (Fig. 3B) in the NK-depleted unvaccinated mice compared to controls.

To investigate whether NA induced protective cytotoxic T cells, we depleted CD8 T cells in NA immunized mice before challenge (depletion confirmed by flow cytometry Supplementary Fig. S1). Similar to the other studies, the NA immunized mice were partially protected; however, there was no impact of CD8 depletion on the partial protection by NA immunization (Fig. 3C). Immunized mice also had significantly less viral RNA after infection than control mice (*P* < 0.001, Fig. 3D). Unlike NK depletion, CD8 depletion in unimmunized mice increased viral load on the challenge (*P* < 0.05), suggesting a role in viral control. The depletion studies overall suggest that the mechanism of protection following NA immunization is likely predominantly antibody-mediated rather than cellular.

### NA immunization does not protect against heterologous virus challenge

**Figure 4:**
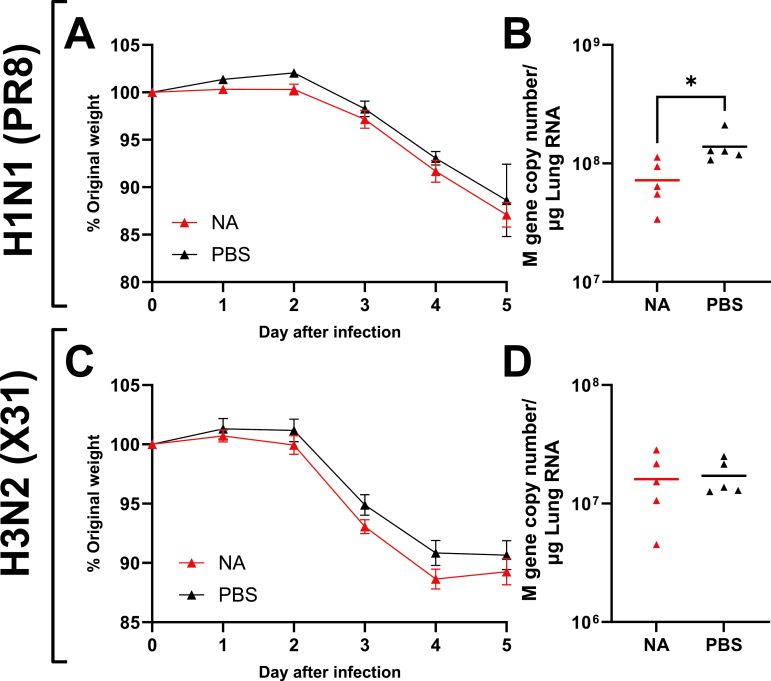
**Immunization with dbDNA encoding NA from Cal09 does not confer heterologous protection.** Female BALB/c mice were intramuscularly immunized with 10 µg dbDNA encoding for NA (from A/California/07/2009) at 0 and 4 weeks, followed by intranasal challenge with either PR8 (H1N1) or X31 (H3N2) strains of influenza virus at 6 weeks. Weight loss was measured after infection (**A** and **C**) and viral load in the lung was measured 5 days after infection (**B** and **D**). *N* = 5 mice per group, points represent individual animals, and lines represent means. Study performed once. Statistical analysis was performed by ANOVA with Tukey test.

One rationale for the inclusion of NA in influenza vaccines is to broaden protection against divergent influenza viruses. Studies have described NA drift rates as being discordant and lower with respect to HA [30, 31], meaning the inclusion of NA may provide some protection in the event of HA drift. To look at the breadth of protection provided by dbDNA encoded NA, we challenged mice with heterologous strains to those used to immunize. Mice were primed and boosted with dbDNA encoding N1 from A/California/07/2009 H1N1 strain and then challenged with either PR8 (A/Puerto Rico/8/1934; H1N1) or X31 (H3N2). The N1 from A/California/07/2009 shares 80.4% sequence identity with A/Puerto Rico/8/1934 and approximately 40% with N2. There was no difference in disease outcome in the immunized compared to the naïve group after PR8 challenge (Fig. 4A). Interestingly, there was a slight but significant reduction in viral load between the immunized and control groups (*P* < 0.05, Fig. 4B). There was no difference in disease severity (Fig. 4C) or viral load (Fig. 4D) following H3N2 infection. This suggests that there may be some marginal benefit of NA against drifted virus within the same strain, but not against different strains.

### Combining NA and HA antigens does not reduce immunogenicity to individual antigens

**Figure 5:**
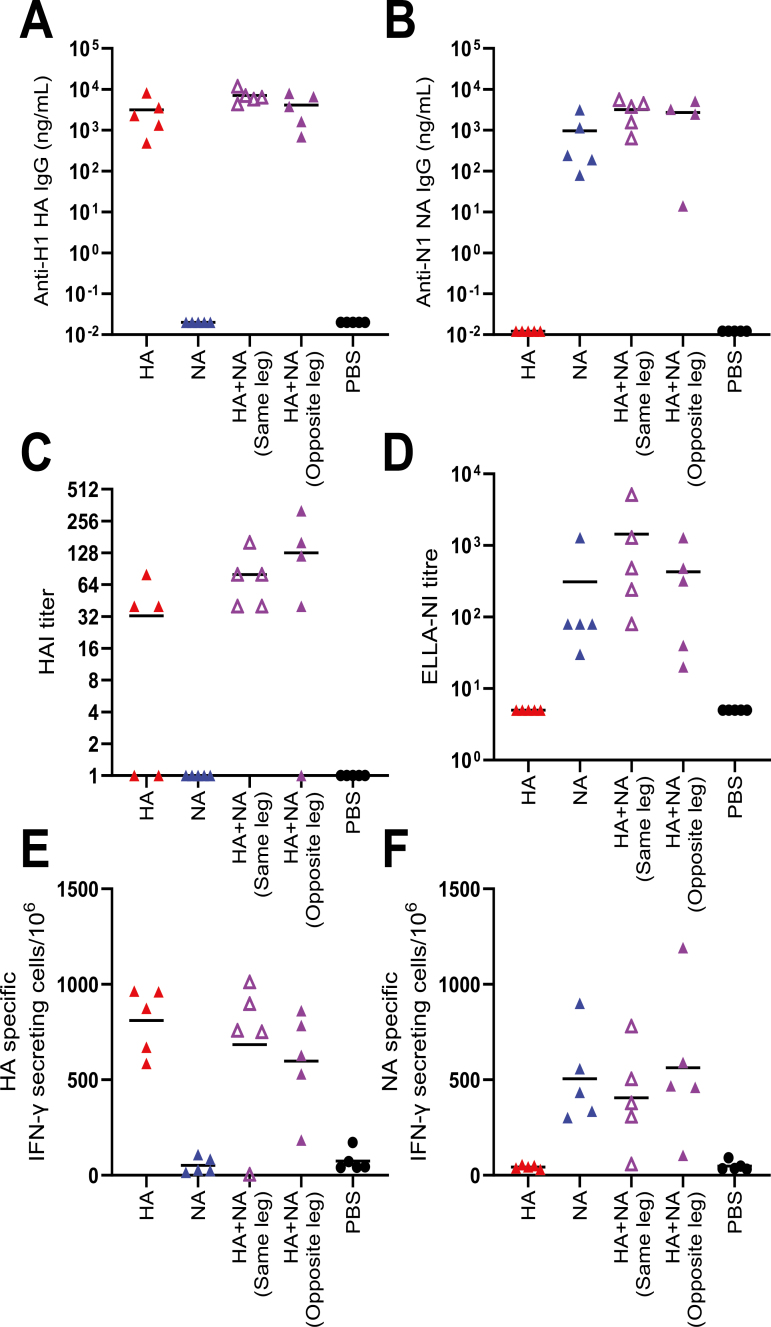
**Co-immunization of NA- and HA-encoding dbDNA is non- detrimental to the resulting immune response.** Mice were immunized with 10μg DTS containing dbDNA encoding HA or NA alone, or co-immunized in the same or different limbs (20 μg total in the co-immunization studies), in a prime-boost regime at 0 and 4 weeks. Serum was collected post-boost at 6 weeks for analysis of HA- (**A**) or NA- (**B**) specific antibody responses by ELISA; HAI (**C**) and ELLA (**D**). Spleens were collected and assessed for HA- (**E**) and NA- (**F**) specific T cells by ELISpot at the same timepoint. *N* = 5 mice per group, points represent individual animals, and lines represent means. Study performed once. Statistical analysis was performed by ANOVA with Tukey test.

One approach to increase the breadth of protection with an influenza vaccine is by delivering HA and NA together in a vaccine [32]. The DNA platform potentially allows for the co-administration of multiple antigens in a single injection, but there is a potential that the expression and immune response to one antigen might dominate over another. One study using a multivalent H5N1 DNA vaccine proposed that favouring cellular responses towards HA led to a decrease in the observed responses against the other vaccine antigens. Having demonstrated the ability of dbDNA vaccines to induce protective HA and NA-mediated responses, co-immunization with dbDNA encoding each antigen was investigated; evaluating delivery in the same injection or simultaneously but in the contralateral flank. Co-immunization did not dampen the antigen-specific responses, either when at the same site or the different leg. The mice received 10μg of each individual dbDNA individually or 20 μg when combined. Anti-HA (Fig. 5A) and anti-NA IgG (Fig. 5B) responses were comparable when antigen was delivered alone or co-immunized. A similar outcome was seen for measurements of antibody function – there was no reduction in HAI (Fig. 5C) or ELLA (Fig. 5D) titres comparing co-immunization of HA and NA with single antigens. Co-immunization also had no detrimental impact on HA-specific (Fig. 5E) or NA-specific (Fig. 5F) IFNγ secreting T cells. This indicates that the co-administration of two antigens from influenza virus in DNA vaccines does not result in immune interference.

## Discussion

In the current study, we explored approaches to optimize a dbDNA vaccine against influenza virus. Initially, we investigated the effect of including DTS on the immunogenicity and protection of dbDNA. Interestingly, there was a difference between the two antigens tested, HA and NA. The DTS had a significant advantage in inducing protective immune responses to influenza infection when combined with the HA antigen. Previously, it has been demonstrated that the incorporation of an SV40-derived DTS into a peptide-encoding DNA delivery vector represented a promising way to improve both DNA transfection and gene expression in slow-dividing cells and dendritic cells [33]. Another study showed that conjugation of the SV40-derived DTS to a MIDGE DNA vector was able to enhance antigen expression, likely due to enhanced nuclear membrane translocation, which ultimately translated into improved humoral and cellular immune responses *in vivo* [34]. As a further example of the utility of DTS incorporation, Le Guen *et al*. recently demonstrated that the incorporation of short nucleotide sequences that interact with the transcription factor NF-κB could provide modest to substantial advantages for plasmid DNA nuclear import *in vivo* [35]. However, we saw no impact on immunogenicity with dbDNA encoded NA, why this was the case is unclear and needs further investigation. Our data suggests that altering non-coding regions of DNA vaccines can have a beneficial impact, but effects vary with different antigens and so optimization needs to be on a case-by-case basis. Having seen that the inclusion of DTS can improve immunogenicity to an HA vaccine, we explored a second approach to improve the overall protective efficacy of the dbDNA influenza vaccine; the incorporation of a second antigen, NA. We saw that dbDNA-encoded NA alone provided partial protection against influenza virus infection, though there was some variability in responses. Protection against viral challenge following immunization with NA-encoding DNA vaccine has been observed in other studies [36, 37]. In another study, DNA encoding HA or NA protected better against challenge virus than mice immunized with M1-, NP-, or NS1 DNA [38]. In ferrets and pigs, an NA-encoding plasmid DNA vaccine was shown to induce antibodies that inhibited the catalytic activity of NA [39]. These data, therefore, suggest that the inclusion of dbDNA NA can have additive protection against influenza vaccine. This is supported by the data showing combining the antigens did not dampen immunogenicity; though we did see some variability in responses, especially in the T cell responses, which is a limitation to the interpretation.

The immunogenicity studies with the NA construct showed it could induce NA binding antibodies but also functional antibodies as measured using the ELLA assay. This assay has recently been validated as a quantitative assay for NA serology [40]. The induction of anti-NA antibodies raised the question of the mechanism of immune protection, whether it was antibody mediated or cellular; this is an important question as we have shown that changing the route of delivery of DNA vaccine can favour a cellular or antibody response [41]. To determine whether cellular responses were protective, we performed NK and CD8 depletion studies in NA immunized mice. We chose to deplete NK cells because they are recognized as being a crucial component of innate immunity protecting against influenza infection [42]. The role of NK cells in vaccine-induced immunity remains unclear with studies evaluating the effect of DNA vaccination on NK cell differentiation being limited, though it was shown that vaccination of rhesus macaques using a DNA vaccine encoding for the SARS-CoV-2 Spike was shown to induce an anti-Spike-dependent NK cell response [43]. We depleted CD8 T cells because they are a major contributor to viral clearance; delayed viral clearance has been observed in mice lacking CD8 T cells [44], and we saw that CD8-depleted unvaccinated mice had a higher viral load. Studies revealed that NA contains several murine CD8 T cell epitopes [45, 46], suggesting they could play a role.

However, our depletion studies suggested that neither CD8 or NK cells are critical for dbDNA NA-mediated protection and so it is likely that antibody is primarily responsible for mediating protection from viral challenge. Though it is possible that CD4 cells are playing a role and we did not explore the impact of depleting this cell type. Previous studies have demonstrated the protective role of anti-NA antibodies induced by DNA immunization. Following a single immunization with an MHC class II-targeting NA DNA vaccine, protection against homologous influenza infection was found to be mediated by NA-inhibiting antibodies [47]. A further study determined that partial cross-protection against avian H5N1 from immunization with human N1-encoding plasmid DNA was mediated by a humoral response [37]. Anti-NA antibodies do not prevent viral entry, instead they limit the spread of the virus, contributing to immunity against influenza in animal models. This is supported by data from human challenge studies conducted in the 1970s, which revealed that anti-NA antibody titres inversely correlated with both viral shedding and observed symptoms.

One consideration for the inclusion of NA in an influenza vaccine is to increase the breadth of protection. Having observed that NA-mediated protection was most likely antibody-mediated, which can potentially be strain-specific, we looked at heterologous infection. Immunization with NA-encoding dbDNA offered some protection against a drifted virus of the same stain but could not protect against a different strain. This protection against drift strains reflects other studies, one study using poly I:C adjuvanted recombinant neuraminidase protein saw robust protection against homologous strains, some subtype-specific cross-reactive antibodies with the potential to protect against drifted seasonal viruses [21]. However, cross-protection was confined within the same subtype, and no intersubtypic protection was identified. Similarly, in a study using NA-encoding plasmid DNA, cross-protection against lethal challenge was only observed against antigenic variants within the same subtype but not against a virus of a different subtype [48]. The addition of an N1-encoding plasmid to an H5 DNA vaccine formulation was found to boost protective efficacy from 75% to 100% when challenged with a distant H5N1 virus [49]. Together, these studies support the inclusion of NA as a way to provide some additional protection.

The presented studies support DNA, especially linear-dsDNA such as dbDNA, as a suitable platform for influenza vaccines due to its flexibility and the ability to incorporate multiple antigens. One limitation for translation into human studies was the use of only female mice as this was a discovery study, further studies on both sexes would be required to finalise preclinical development before proceeding to GLP safety and clinical studies. Another area that needs optimization for moving into human studies is the delivery of the DNA; in these studies, we used electroporation, which though it has been shown to induce immune responses in phase I clinical trials [50] with some DNA cancer studies progressing to phase III [51, 52], is not practical for wider deployment. An alternative consideration is the formulation of the DNA to increase uptake, which had such a potent effect on RNA vaccines for COVID-19 [53]. Further exploration of different aspects of DNA vaccines will help deliver on the promise of the platform.

## Supplementary Material

kyad030_suppl_Supplementary_Figures_S1

## Data Availability

Numerical data from graphs are available on request to the corresponding author.

## Abbreviations

APCs: antigen-presenting cells
dbDNA: Doggybone™ DNA
DTS: DNA targeting sequences
HA: haemagglutinin
HAI: haemagglutination inhibition
NA: neuraminidase
NLS: nucler localization signal
PEG: polyethylene glycol
SPF: specific-pathogen-free
