# Supplementary material for: Optimizing a linear ‘Doggybone’ DNA vaccine for influenza virus through the incorporation of DNA targeting sequences and neuraminidase antigen
Source: Discov Immunol. 2024 Jan 3;3(1):kyad030. doi: 10.1093/discim/kyad030 (PMC10917164; doi:10.1093/discim/kyad030)
Supplement: kyad030_suppl_Supplementary_Figures_S1 [file kyad030_suppl_Supplementary_Figures_S1.pptx]

## Slide 1
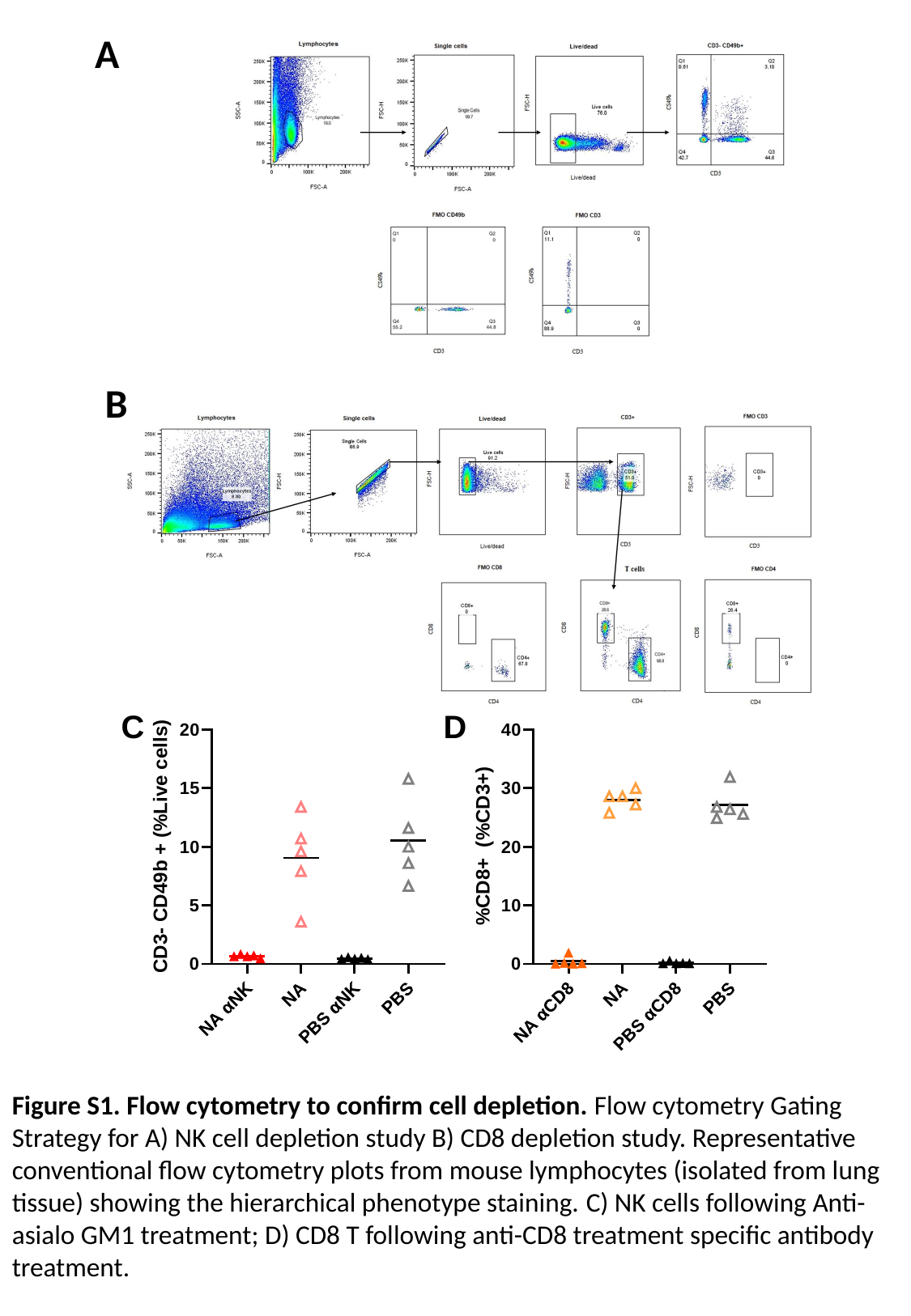

A
B
Figure S1. Flow cytometry to confirm cell depletion. Flow cytometry Gating Strategy for A) NK cell depletion study B) CD8 depletion study. Representative conventional flow cytometry plots from mouse lymphocytes (isolated from lung tissue) showing the hierarchical phenotype staining. C) NK cells following Anti-asialo GM1 treatment; D) CD8 T following anti-CD8 treatment specific antibody treatment.
